# Occurrence and Removal of Triazine Herbicides during Wastewater Treatment Processes and Their Environmental Impact on Aquatic Life

**DOI:** 10.3390/ijerph19084557

**Published:** 2022-04-10

**Authors:** Meng Wang, Jiapei Lv, Haowei Deng, Qiong Liu, Shuxuan Liang

**Affiliations:** 1 Key Laboratory of Analytical Science and Technology of Hebei Province, College of Chemistry and Environmental Science, Hebei University, Baoding 071002, China; wangm1022@126.com (M.W.); dhw2028@163.com (H.D.); lq15030106275@163.com (Q.L.); 2 State Key Laboratory of Environmental Criteria and Risk Assessment, Chinese Research Academy of Environmental Sciences, Beijing 100012, China; lvjp@craes.org.cn

**Keywords:** triazine pesticides, removal, risk evaluation, wastewater treatment plants

## Abstract

Wastewater treatment plants (WWTPs) represent a major point source for pesticide residue entry to aquatic environment and may threaten ecosystems and biodiversity in urban area. Triazine herbicides should be paid attention to for their ubiquitous occurrence in the environment and long-term residue. The present study aimed to quantify eleven compounds of triazine herbicides during wastewater treatment processes. The solid phase extraction and gas-chromatography mass spectrometry (GC-MS) determination method were developed to identify the target herbicides with approving sensitivity. The pollution levels, removal rates of eleven triazine herbicides along five different treatment stages in WWTP were investigated. The results showed that three herbicides including atrazine, simetryn and prometryn were detected. Their concentrations in influent were among 28.79 to 104.60 ng/L. Their total removal rates from influent to effluent were 14.92%, 10.79% and 4.41%, respectively indicating that they were difficult to be effectively remove during wastewater treatment. Regarding the negative impact of triazine herbicides discharged from WWTPs on downstream water quality and aquatic life, the environmental risks were assessed by calculating the Environmental Relevance of Pesticides from Wastewater Treatment Plants Index (*ERPWI*) and water cycle spreading index (*WCSI*). The risk assessment results denoted the possible high risks for atrazine and simetryn to alage, and simetryn concurrently posed a high risk for the daphnia, while prometryn was at medium risk to alage. Atrazine and simetryn in effluent posed high risk for algae, meanwhile, simetryn had high risk for Daphnia. These results suggested a possible threat to the aquatic environment, rendering in this way the *ERPWI* method as a useful assessment tool. Further extensive study is needed for atrazine and simetryn in order to better understand their migration mechanism in aquatic environment.

## 1. Introduction

Herbicide is a powerful weed-controlling substance and has been extensively utilized in revolutionizing agriculture to increase crop yields [1]. Their consumption account for more than 50% of the agricultural market. Triazine herbicides, known as a milestone in the development of weed control technology, have been registered in more than 100 countries and are key to the production of more than 50 crops. They have been widely used to prevent weed growth in both agricultural production and urban utility management [2,3,4]. However, most triazine herbicides possess long residual activity and low volatility [5]. Their wide application may accumulate in soil and crops, even pollute rivers by surface runoff [6,7]. There are reports of triazine exposure resulting in mutagenic and hormone-disrupting effects DNA comet formation on aquatic organisms [8,9,10] and impact the aquatic environment [11,12]. Humans also display toxicity following exposure to the herbicides through ingestion, inhalation, or dermal contact [13].

The global coastal pollution status of herbicides and their negative impact on ecosystem in natural environmental concentrations have caused widespread concern [14]. Some rather herbicides have been frequently detected in freshwater environments (rivers, lakes, pond, etc.) worldwide, with the concentrations being around 1 to 104 ng/L [15,16]. Ultimately, coastal pollution of herbicides and their negative impact on marine life also been reported in recent years [12,17]. Although a variety of herbicide residuals have been identified in freshwater or seawater, studies on pesticides elimination during wastewater treatment are relatively rare since these substances are typically considered of agricultural rather than of freshwater environment [18]. Wastewater treatment plants (WWTPs) receive the municipal domestic wastewater of all residents, including the cleaning of fruits and vegetables, adsorption of atmospheric sedimentation or other irregular use. The residual herbicides are indirectly or directly discharged into WWTPs. Some compounds are removed in the precipitation, biochemical, and disinfection units in WWTPs, but the refractory compounds cannot be removed and subsequently enter the water environment with the effluent, which probably poses a threat to river water quality and aquatic life [19,20,21]. A study of the contribution of WWTPs to pesticide toxicity in downstream rivers found that herbicides contributed the most to total pesticide toxicity in water compared to fungicides and insecticides [22]. WWTPs represent a major point source for pesticide residue entry to aquatic environment and may threaten freshwater ecosystems and biodiversity urban aera. Therefore, it is of significant issue to study the existence of herbicides in WWTPs. To the best of our knowledge, the distribution and removal of triazine herbicides in WWTPs have not been reported so far.

The introduction of these herbicide in environment presents a risk to the aquatic environment. Besides the residual concentration and removal, there is an urgent need to clarify the hazards of herbicides, and most importantly, uncover their current threats to ecosystems in downstream of municipal wastewater outlet. Ecological risk assessment is given as a function of environmental exposure and ecotoxicological effects. A risk assessment of NP was usually actualized by the calculation of risk quotient (RQ). This is usually expressed as the ratio of the predicted environmental concentration (PEC) to predicted no-effect concentration (PNEC) [23,24]. With regard to WWTPs, the degree of removal to the contaminants is also an important aspect in terms of risk as it helps identify which are the most recalcitrant compounds and hence the most persistent and relevant herbicides to the aquatic environment. Thus, the Environmental Relevance of Pesticides from Wastewater treatment plants Index (*ERPWI*) is proposed in this work. This index embraces not only the occurrence and removal of pesticides in wastewater treatment installations, but also the toxicity of each pesticide against three aquatic organisms including algae, daphnia and fish [18]. In addition, a water cycle spreading index (*WCSI*) was cited by calculation the quotient of the effluent concentration of a compound by its relative removal in WWTP in this work. This index provides a measure for the potential of a concerned compound to spread along a partially closed water cycle after discharge with municipal wastewater [25].

The present study uses a combination of solid phase extraction (SPE) and gas chromatography-mass spectrometry (GC-MS) as an analytical tool for the screening of a class of the most widely used triazine herbicide residues in wastewaters in each treatment unit in a WWTP. The distribution and risk of these typical herbicides were systematically surveyed. The objectives of this study aimed to (1) apply the developed quantitative and qualitative methodology for the analysis of the residual levels of triazine herbicides; (2) investigate the removal rates of each target compound in the WWTP; (3) the environmental relevance of this source of triazine herbicides in effluent to the aquatic environment for three taxonomic groups (algae, daphnia and fish). To the best of our knowledge, the distribution and removal of triazine herbicides in each treatment unit in WWTPs have not been reported so far. The novelty of this work lies, on the other hand, the environmental relevance of WWTP source of triazine herbicides in effluent to the aquatic environment for three taxonomic groups (algae, daphnia and fish) is first study reporting. This study is intensely subjected to environmental risk substances and the aim of this work has been to provide a better understanding of the effect of pesticides in urban area.

## 2. Materials and Methods

### 2.1. Chemicals and Reagents

Standard solutions of mixed 11 triazine herbicides in hexane/acetone were purchased from Alta Scientific Co., Ltd. (Tianjin, China). The standard solution was diluted by acetone to 10 mg/L as stock solution and stored in the dark at −20 °C. Before being used, the solution was dilute to a suitable concentration. Chromatography-grade acetone and analytical-grade methanol were supplied by Tianjin Kermel Chemical Reagent Co., Ltd. (Tianjin, China).

### 2.2. Study Area and Sampling

**Figure 1:**
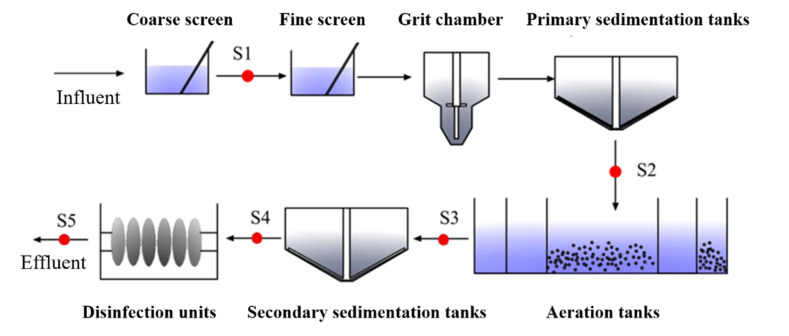
T Schematic diagram of wastewater treatment process of WWTP and sampling points (S1–S5).

The wastewater treatment plant (WWTP) in this study is located on the Baoding city, Hebei province, China. The WWTP studied receive mainly domestic wastewater and urban runoff. The maximum processing capacity of the WWTP is 160,000 m^3^/d. The WWTP process is schematically shown in Figure 1. The WWTP consists of coarse grit, fine screen, grit chamber, primary sedimentation tanks, biological treatment, secondary sedimentation tank, and disinfection units. Phoredox process is used as the core biological treatment process in the WWTP to ensure the effective removal of degradable organics, total nitrogen (TN) and total phosphorus (TP). The Phoredox process is separated into five compartments in sequence to create anaerobic, first anoxic, first aerobic, second anoxic, and second aerobic.

Five sampling points were set in different water treatment units in this study (shown in Figure 1). Wastewater samples were collected from every sampling point of the WWTP. The sampling was programed to collect 2 L of wastewater every 30 min during 24 h using discrete sampling, and took into consideration the hydraulic retention time. The samples were transported to the laboratory. Immediately upon reception each sample was filtered through 0.45 μm aqueous membrane (Whatman, Maidstone, UK) and then stored at −20 °C.

### 2.3. Analytical Method

The solid phase extraction (SPE) was used as sample preparation according to the previous work with some modifications. Briefly, 1 L water sample were enriched by C18 cartridges (500 mg sorbent/6 mL cartridge), which previously activated by passing 10 mL methanol and 10 mL ultra-water. The cartridges were washed by 5 mL ultra-water and dried at −25 kPa for 30 min, then target analytes were eluted by passing 10 mL methanol by inches. Eluates were evaporated to 2 mL and blown to nearly dry with highly-pure nitrogen. The eluates were reconstituted with 1 mL acetone.

The analysis was carried out using gas-chromatography mass spectrometry (GC-MS). GC-MS System included an Agilent G 4513A auto-injector and an Agilent Technologies 7890B gas chromatograph coupled with a 5977 Agilent mass detector. The chromatography column used for analyte separation was a HP-5MS column, 30 m × 0.25 mm with 0.25 μm film thickness of (5%–phenyl)-methylpolysiloxane phase from Agilent (Santa Clara, CA, USA). The inlet temperature was kept to 265 °C and the injection volume was set at 1 μL in splitless mode. For the analysis, a gradually increasing temperature was performed according to the following program: initial temperature 80 °C for 2 min, ramped up to 150 °C at a rate of 25 °C/minute and maintained for 9 min, followed up to 230 °C at the same rate then hold at 230 °C for 2 min. MS ionization mode was electron ionization. An ion source temperature set at 230 °C and an interface temperature of 280 °C were operated. Quantitative analysis was performed using selected ion-monitoring mode (SIM) on GC-MS.

### 2.4. Quality Control and Statistical Analysis

Prior and after each sampling batch, instrument blanks are analyzed and the calibration curve was injected. Repeat three times for each sample and one procedural blank was analyzed for every three samples.

The experimental data were collected treated in Agilent Mass Hunter workstation. All quantitative data were compiled in a database and further subjected to statistical evaluation using SPSS 25 from IBM company (Chicago, IL, USA) for statistical analysis. Origin 9.5 (Northampton, MA, USA) was used to plot the graphs.

### 2.5. Risk Assessment

Risk levels could be quantified the environmental impact among different chemical pollutants to facilitate comparison. In this study, both methods of *ERPWI* and *WCSI* were used for risk assessment. *ERPWI* considers the occurrence and removal of triazine herbicides in WWTPs, and also their toxicity against three aquatic microorganisms: alage, fish, daphnia. *ERPWI* was employed in the following calculation according to the Equation (1):

(1) $$ERPWI=TU_{P}\times S_{rem}\times 1000$$

where *TU_p_* = herbicide concentration in effluent (ng/L)/end point (LC_50_, EC_50_) (ng/L), *S_rem_* is the removal score based on the removal rate of pesticides in WWTPs.

*WCSI* was estimated based on the ratio of effluent concentration and the removal rate of WWTPs. Reemtsma et al. [25] put forward polar pollutants into the water cycle of urban wastewater, taking into account the concentration of each pollutant in wastewater and the relative elimination during the treatment process without considering the toxicity of the compounds to the aquatic organisms. The purpose of *WCSI* is to assess the potential of compounds to diffuse in the aquatic environment. *WCSI* can be calculated as follows Equation (2):

(2) $$WCSI=\frac{C_{in}\times C_{out}}{C_{in}-C_{out}}$$

where *C_in_* is the herbicide concentration in influent (ng/L) and *C_out_* is the herbicide concentration in effluent (ng/L) in WWTPs.

## 3. Results

### 3.1. Performance of the Analytical Method

After a series of optimization experiments, sample pretreatment was optimized that a single extraction procedure using SPE would allow the multi-residue determination of selected 11 triazine herbicides in wastewaters. This sample pretreatment step was combined with the GC-MS using SIM mode for the qualification and quantification of the target analytes. The analytical methods have been accurately verified in water samples before determination. The range of calibration curves were 0.01–1 mg/L with good linear fitting. The recovery rates were between 59% and 116%, and relative standard deviations (RSD) ranged 2–11%. The limits of detections (LOD), as shown in Table 1, were in the range of 15–47 ng/L. The calibration curve was detected before each sampling batch. A blank procedure was carried every 5 samples.

### 3.2. Individual Triazine Herbicide Levels

The physicochemical parameters of wastewater including suspended solid (SS), chemical oxygen demand (COD), total nitrogen (TN) and total phosphorus (TP) were determined. Table S1 shows the detailed characteristics of different sample positions of WWTP during the study.

The average concentrations of the individual triazine herbicides in the water samples is shown in Table 2 distinguishing among wastewater from different units. The comparison of the individual herbicide median levels in WWTPs is showed in Table S2. As it can be seen in Table 2 and Table S2, the most ubiquitous pesticides were atrazine, simetryn and prometryn, detected in each sample, while other 8 herbicides, including atratone, simazine, prometon, propazine, terbuthylazine, secbumeton, ametryn, terbutryn, were not detected in any samples. The maximum individual concentration was observed for atrazine (130.75 ng/L in secondary sedimentation tank), followed by simetryn (102.08 ng/L in end aeration).

The highest concentration of herbicide detected was atrazine. It’s concentration in influent and effluent were 104.59 and 89.04 ng/L respectively. Rimayi et al. [26] detected six triazine herbicide abundance concentrations of atrazine, simazine, propazine, ametryn and prometryn in South Africa. They also found atrazine was the most abundant triazine herbicide (>130 ng/L). Wittmer et al. [27] aimed to improving our understanding of the influence of pesticides in surface water on urban and agricultural land. Their results revealed the elevated background concentrations throughout the year, indicating a constant household source. Atrazine is a widely used herbicide that can be applied before and after planting to control broadleaf and grassy weeds. It is not only used in primarily in agriculture, with the greatest use on corn, sorghum, and sugarcane. To a lesser extent, it is also used on residential lawns [28]. Atrazine is the triazine herbicide with the highest sales, accounting for one-third of this kind of herbicide. From the perspective on dosage and residue, atrazine residual should be paid more closer attention.

### 3.3. Removal of Herbicides in the WWTP

The performance of WWTP can be reflected based on the removal rate of pollutants in wastewater. The removal rate of triazine in wastewater in this study is shown in Figure S1. This figure clearly showed the poor overall removal of herbicides in the WWTP. Atrazine has the highest removal rate, in which about 14.92% of the influent was removed. The removal rate of simetryn was 10.79%, while removal rate of prometryn was the lowest at 4.41%.

Previous researches on the removal of pesticides in WWTPs have also suggested poor performance. Campo et al. [29] investigated the occurrence and removal of more than 40 pesticides in 16 WWTPs and the result showed 64% of the pesticides targeted were not eliminated in tertiary treatment. The removal rate of triazine herbicides were −570–91%. Köck-Schulmeyer et al. [18] investigated the occurrence and removal of 22 pesticides (atrazine, simazine, terbuthylazine included) in three WWTPs. They found atrazine was the poorest removal among various compounds, followed by simazine and terbuthylazine. Furthermore, the concentration of pesticide effluent is higher than the concentration of influent. The probable reasons for the low or negative removal rates were desorption and hydrolysis of particulate matter, degradation and transformation of micropollutants during the biochemical process.

### 3.4. Risk Assessment

Wastewater is discharged into downstream rivers after being treated by WWTPs. Unremoved pollutants have a certain contribution to the river contamination, which further affects the aquatic environment. Different pesticides of the same concentration have different degrees of harm to different aquatic organisms. Therefore, the assessment method took into account the toxic effects on the aquatic environment. In this study, *ERPWI* comprehensively considered the removal rate of pesticides by WWTPs and the toxicity of aquatic organisms. Table 3 lists the LC_50_ or EC_50_ values of the target pesticides on algae, daphnia and fish. Table 4 shows the removal scores (*S_rem_*) at different removal rate intervals, and the classification of their risk levels according to *ERPWI* values.

**Figure 2:**
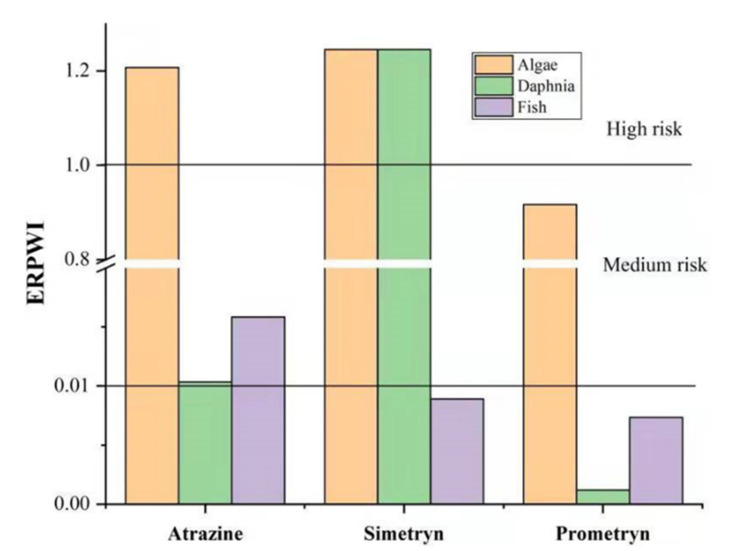
*ERPWI* values of the herbicides on the three aquatic organisms.

The *ERPWI* values for the target herbicides were shown in Figure 2. In the case of algae, *ERPWI* value of atrazine and simetryn were at a high risk level, while prometryn was at medium risk level. The reason of high value for atrazine and simetryn calculated could be attributed to the higher toxicity for alage. The calculated *ERPWI* indicated a high risk level for simetryn, a low risk level for atrazine, and a negligible risk level for prometryn in the daphnia. The risk level for simetryn could be attributed to its higher toxicity than other herbicides. The *ERPWI* for atrazine in the case of fish is at a low risk level, while simetryn and prometryn are at negligible risk levels, which could be attributed to the low concentration in the effluent and little toxicity for fish. Köck-Schulmeyer et al. [18] summarized that the calculated *ERPWI* value of triazine herbicides was at low and medium risk, which was consistent with the results of this study.

The *WCSI* values for the target herbicides were shown in Figure S2. As shown in Figure S2, the calculated *WCSI* values of the three triazine herbicides were all at a high level, which was because of the low removal rate in WWTPs. The lower removal rates of herbicides may be due to the difficult degradation. The moderate effluent concentration but poorly removal compound in the WWTP is considered more harmful, compared to the same concentration and extensively removal compound. The higher the *WCSI*, the more likely the pollutant is to diffuse in the water environment and water cycle [25]. It is worth noting that *WCSI* was mostly influenced by the normal operation of the WWTPs. Therefore, the high *WCSI* value may be caused by some working faults of the WWTPs or the composition of influent wastewater may not change much [30].

## 4. Conclusions

The pollution of triazine herbicides to the aquatic environment in urban areas has already attracted universal attention. Monitoring of the occurrence of triazines in wastewater sources and their removal in wastewater treatment plant is of unquestionable importance. We attempted to systematically surveyed the occurrence of triazine pesticides in wastewater sources and the removal rates during each unit in wastewater treatment plants. The Environmental Relevance of Pesticides from Wastewater treatment plants Index and water cycle spreading index are used for the risk evaluation. The proposed methods took into account the concentration in wastewater, removal rate in WWTPs, and toxicity to aquatic organisms. The results of this study indicated that the present treatment of wastewater had the disadvantages of low removal rates from 4.41% to 14.92%. And the residual concentrations of triazine herbicides in wastewaters of the municipal wastewater treatment plant had been beyond the “high risks” for atrazine and simetryn on algae or daphnia (*ERPWI* > 1). These results suggested that triazine herbicides are becoming a new threat to the primary productivity and urban ecological ecosystems. The hazard may become more severe with the increase of herbicide application. Ecological risk assessments could provide data support for management decisions. To maintain aquatic ecosystem stability and human health, pesticide regulations should be strictly managed, monitoring continuously of wastewater and strengthening of quality control are recommended.

## Figures and Tables

**Table 1:** Recoveries and RSDs of the selected pesticides.

Compound	Rt (min)	R^2^	LOD (ng/L)	Concentration (ng/L)	Recovery (%)	RSD (%)
Atratone	15.82	0.993	34	500	98.62	3.10
				50	64.42	2.20
Simazine	15.93	0.997	38	500	62.46	9.16
				50	80.57	4.08
Prometon	15.99	0.997	26	500	99.95	2.24
				50	95.23	6.07
Atrazine	16.07	0.999	23	500	88.40	7.14
				50	87.66	5.68
Propazine	16.18	0.999	20	500	93.68	5.36
				50	80.72	8.32
Terbuthylazine	16.39	0.999	18	500	93.94	6.98
				50	80.69	10.00
Secbumeton	16.77	0.996	19	500	116.80	2.42
				50	104.02	6.67
Simetryn	17.57	0.997	21	500	86.72	11.13
				50	100.69	11.45
Ametryn	17.65	0.998	45	500	92.39	9.33
				50	59.00	11.68
Prometryn	17.72	0.999	47	500	88.07	4.97
				50	70.50	11.76
Terbutryn	17.94	0.998	15	500	96.04	5.75
				50	102.38	11.06

**Table 2:** The detected concentrations of the triazine herbicides in different units.

Sampling Location	Concentration (ng/L)		
	Atrazine	Simetryn	Prometryn
Influent	104.59 ± 5.03	87.23 ± 1.00	28.79± 1.90
Primary sedimentation tank	121.89 ± 2.85	97.27 ± 4.36	29.90 ± 1.34
End aeration	114.63 ± 4.51	102.08 ± 7.22	29.86 ± 2.37
Secondary sedimentation tank	130.75 ± 2.46	102.87 ± 5.78	31.08 ± 1.58
Effluent	89.04 ± 5.95	77.83 ± 1.97	27.50 ± 1.40

**Table 3:** EC_50_ and LC_50_ of the herbicides for three aquatic organisms.

Herbicides	EC_50_ (mg/L)	EC_50_ (mg/L)	LC_50_ (mg/L)
	Algae ^1^	Daphnia ^2^	Fish ^3^
Atrazine ^a^	0.059	6.9	4.5
Simetryn ^b^	0.05	0.05	7
Prometryn ^b^	0.024	18.59	3

^a^ Köck-Schulmeyer et al., 2013 [18]; ^b^ PAN Pesticides Database—Chemical Toxicity Studies on Aquatic Organisms. ^1^—72 h; ^2^—48 h; ^3^—96 h.

**Table 4:** Removal scores and *ERPWI* classification.

Removal Rate (%)	*S_rem_*	*ERPWI*	Risk Level
75–100	0.2	>10	Very high
50–75	0.4	1–10	High
25–50	0.6	0.01–1	Medium
0–25	0.8	0.001–0.01	Low
<0	1.0	<0.001	Negligible

## Data Availability

Not applicable.

## Supplementary Materials

The following supporting information can be downloaded at: https://www.mdpi.com/article/10.3390/ijerph19084557/s1, Figure S1. Removal of herbicides in the municipal wastewater treatment plant. Figure S2. WCSI values of the herbicides in the WWTP effluent. Table S1. Characteristics of wastewater in different units. Table S2. Comparison of the individual herbicide concentrations in wastewater.

## References

[1] Zheng S., He M., Chen B., Hu B. (2020). Porous aromatic framework coated stir bar sorptive extraction coupled with high performance liquid chromatography for the analysis of triazine herbicides in maize samples. J. Chromatogr. A.

[2] Liu C., Dou X., Zhang L., Li Q., Qin J., Duan Y., Yang M. (2018). Determination of triazine herbicides and their metabolites in multiple medicinal parts of traditional Chinese medicines using streamlined pretreatment and UFLC-ESI-MS/MS. Chemosphere.

[3] Zhang F., Zhao Q., Yan X., Li H., Zhang P., Wang L., Zhou T., Li Y., Ding L. (2016). Rapid preparation of expanded graphite by microwave irradiation for the extraction of triazine herbicides in milk samples. Food Chem..

[4] Zhou J., Chen J., Cheng Y., Li D., Hu F., Li H. (2009). Determination of Prometryne in water and soil by HPLC-UV using cloud-point extraction. Talanta.

[5] Elmore C.L., Lange A.H. (2008). The Triazine Herbicides.

[6] Du Preez L.H., Jansen Van Rensburg P.J., Jooste A.M., Carr J.A., Giesy J.P., Gross T.S., Kendall R.J., Smith E.E., Van Der Kraak G., Solomon K.R. (2005). Seasonal exposures to triazine and other pesticides in surface waters in the western Highveld corn-production region in South Africa. Environ. Pollut..

[7] Solomon K.R., Carr J.A., Du Preez L.H., Giesy J.P., Kendall R.J., Smith E.E., Van Der Kraak G.J. (2008). Effects of atrazine on fish, amphibians, and aquatic reptiles: A critical review. Crit. Rev. Toxicol..

[8] Adeyemi J.A., Martins-Junior A.C., Barbosa F. (2015). Teratogenicity, genotoxicity and oxidative stress in zebrafish embryos (Danio rerio) co-exposed to arsenic and atrazine. Comp. Biochem. Physiol. Part C Toxicol. Pharmacol..

[9] McLachlan J.A. (2016). Environmental signaling: From environmental estrogens to endocrine- disrupting chemicals and beyond. Andrology.

[10] Van Der Kraak G.J., Hosmer A.J., Hanson M.L., Kloas W., Solomon K.R. (2014). Effects of atrazine in fish, amphibians, and reptiles: An analysis based on quantitative weight of evidence. Crit. Rev. Toxicol..

[11] Wirbisky S.E., Freeman J.L. (2017). Atrazine exposure elicits copy number alterations in the zebrafish genome. Comp. Biochem. Physiol. Part C Toxicol. Pharmacol..

[12] Yang L., Li H., Zhang Y., Jiao N. (2019). Environmental risk assessment of triazine herbicides in the Bohai Sea and the Yellow Sea and their toxicity to phytoplankton at environmental concentrations. Environ. Int..

[13] Simpkins J.W., Swenberg J.A., Weiss N., Brusick D., Eldridge J.C., Stevens J.T., Handa R.J., Hovey R.C., Plant T.M., Pastoor T.P. (2011). Atrazine and breast cancer: A framework assessment of the toxicological and epidemiological evidence. Toxicol. Sci..

[14] Huang H.F., Liu H.F., Xiong S., Zeng F.M., Bu J.W., Zhang B., Liu W., Zhou H., Qi S.H., Xu L. (2021). Rapid transport of organochlorine pesticides (OCPs) in multimedia environment from karst area. Sci. Total Environ..

[15] Salla G.B.F., Bracht L., Parizotto A.V., Comar J.F., Peralta R.M., Bracht F., Bracht A. (2019). Kinetics of the metabolic effects, distribution spaces and lipid-bilayer affinities of the organo-chlorinated herbicides 2, 4-D and picloram in the liver. Toxicol. Lett..

[16] Herrero-Hernández E., Rodríguez-Cruz M.S., Pose-Juan E., Sánchez-González S., Andrades M.S., Sánchez-Martín M.J. (2017). Seasonal distribution of herbicide and insecticide residues in the water resources of the vineyard region of La Rioja (Spain). Sci. Total Environ..

[17] Fisch K., Brockmeyer B., Gerwinski W., Schulz-Bull D.E., Theobald N. (2021). Seasonal variability, long-term distribution (2001–2014), and risk assessment of polar organic micropollutants in the Baltic Sea. Environ. Sci. Pollut. Res..

[18] Köck-Schulmeyer M., Villagrasa M., López de Alda M., Céspedes-Sánchez R., Ventura F., Barceló D. (2013). Occurrence and behavior of pesticides in wastewater treatment plants and their environmental impact. Sci. Total Environ..

[19] Loos R., Carvalho R., António D.C., Comero S., Locoro G., Tavazzi S., Paracchini B., Ghiani M., Lettieri T., Blaha L. (2013). EU-wide monitoring survey on emerging polar organic contaminants in wastewater treatment plant effluents. Water Res..

[20] Wang Y., Gao W., Wang Y., Jiang G. (2019). Suspect screening analysis of the occurrence and removal of micropollutants by GC-QTOF MS during wastewater treatment processes. J. Hazard Mater..

[21] Xiao S., Hu S., Zhang Y., Zhao X., Pan W. (2018). Influence of sewage treatment plant effluent discharge into multipurpose river on its water quality: A quantitative health risk assessment of Cryptosporidium and Giardia. Environ. Pollut..

[22] Le T.D.H., Scharmüller A., Kattwinkel M., Kühne R., Schüürmann G., Schäfer R.B. (2017). Contribution of waste water treatment plants to pesticide toxicity in agriculture catchments. Ecotoxicol. Environ. Saf..

[23] Kapsi M., Tsoutsi C., Paschalidou A., Albanis T. (2019). Environmental monitoring and risk assessment of pesticide residues in surface waters of the Louros River (N.W. Greece). Sci. Total Environ..

[24] Reis E.O., Santos L.V.S., Lange L.C. (2021). Prioritization and environmental risk assessment of pharmaceuticals mixtures from Brazilian surface waters. Environ. Pollut..

[25] Reemtsma T., Weiss S., Mueller J., Petrovic M., González S., Barcelo D., Ventura F., Knepper T.P. (2006). Polar pollutants entry into the water cycle by municipal wastewater: A European perspective. Environ. Sci. Technol..

[26] Rimayi C., Odusanya D., Weiss J.M., de Boer J., Chimuka L. (2018). Seasonal variation of chloro-s-triazines in the Hartbeespoort Dam catchment, South Africa. Sci. Total Environ..

[27] Wittmer I.K., Bader H.P., Scheidegger R., Singer H., Lück A., Hanke I., Carlsson C., Stamm C. (2010). Significance of urban and agricultural land use for biocide and pesticide dynamics in surface waters. Water Res..

[28] U.S. Environmental Protection Agency (USEPA) (2020). Pesticide Registration Review: Proposed Interim Decisions for Several Triazines. Fed. Regist..

[29] Campo J., Masiá A., Blasco C., Picó Y. (2013). Occurrence and removal efficiency of pesticides in sewage treatment plants of four Mediterranean River Basins. J. Hazard Mater..

[30] Stamatis N., Hela D., Konstantinou I. (2010). Occurrence and removal of fungicides in municipal sewage treatment plant. J. Hazard Mater..

