# Supplementary material for: Occurrence and Removal of Triazine Herbicides during Wastewater Treatment Processes and Their Environmental Impact on Aquatic Life
Source: Int J Environ Res Public Health. 2022 Apr 10;19(8):4557. doi: 10.3390/ijerph19084557 (PMC9024823; doi:10.3390/ijerph19084557)
Supplement: Supplementary file 1 [file ijerph-19-04557-s001.zip › ijerph-1640448-supplementary.pdf]

# Supplementary Material

## Occurrence and removal of triazine herbicides during wastewater treatment processes and their environmental impact on aquatic life

Meng Wang[1], Jiapei Lv[2], Haowei Deng[1], Qiong Liu[1], Shuxuan Liang[1,*]

[1] Key Laboratory of Analytical Science and Technology of Hebei Province,

College of Chemistry and Environmental Science, Hebei University, Baoding 071002, China;

[2] State Key Laboratory of Environmental Criteria and Risk Assessment,

Chinese Research Academy of Environmental Sciences, Beijing 100012, China

* Correspondence: liangsx168@126.com

## Figure captions

**Figure S1.** Removal of herbicides in the municipal wastewater treatment plant.

**Figure S2.** WCSI values of the herbicides in the WWTP effluent.

## Table legends

**Table S1.** Characteristics of wastewater in different units (n=3).

**Table S2.** Comparison of the individual herbicide concentrations in wastewater.

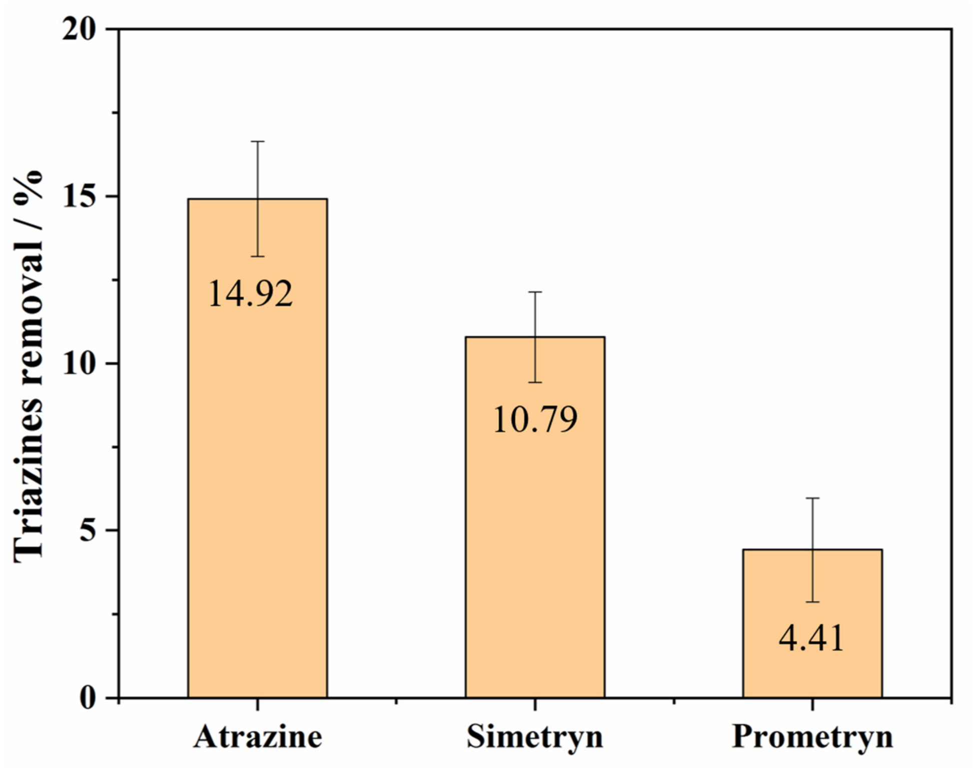


**Fig. S1**. Removal of herbicides in the municipal wastewater treatment plant.

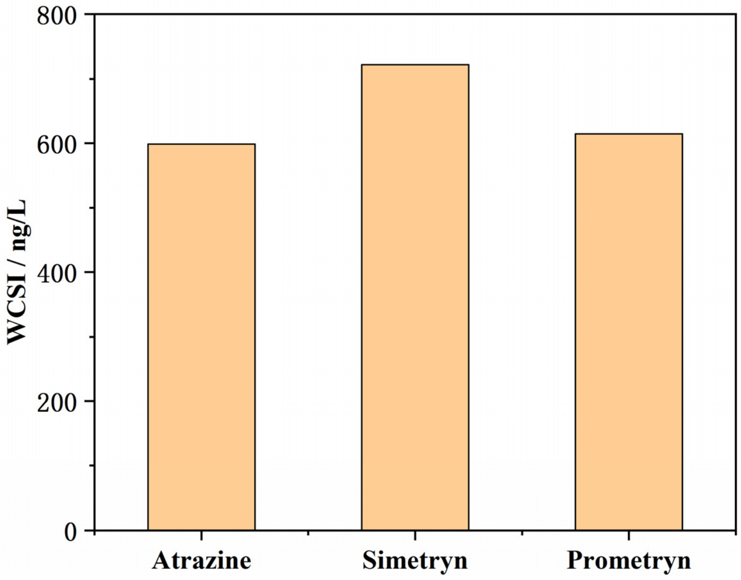


**Fig. S2**. WCSI values of the herbicides in the WWTP effluent.

**Table S1.** Characteristics of wastewater in different units (n=3).

| Parameter | Influent | Primary sedimentation tank | End aeration | Secondary sedimentation tank | Effluent |
|---|---|---|---|---|---|
| SS (mg/L) | 353.67±3.21 | 283.00±11.79 | 3656.67±30.55 | 4.10±0.2 | 3.33±0.15 |
| COD (mg/L) | 232.97±0.97 | 121.13±2.58 | 36.93±0.70 | 32.61±1.19 | 31.63±1.15 |
| TN (mg/L) | 46.77±1.62 | 45.59±2.28 | 6.89±0.03 | 7.07±0.23 | 6.52±0.11 |
| TP (mg/L) | 6.17±0.35 | 5.60±0.33 | 0.35±0.02 | 0.26±0.02 | 0.23±0.03 |
| T (°C) | 28.57±0.06 | 28.47±0.06 | 28.63±0.06 | 28.57±0.06 | 28.27±0.06 |

**Table S2**. Comparison of the individual herbicide concentrations in wastewater.

| Herbicides | Concentration (ng/L) | | | |
|---|---|---|---|---|
| | 2010[(a)] | 2011[(a)] | 2013[(b)] | This study |
| Atrazine | 3.90-27.44 | 7.20-36.90 | 1.23-7.4 | 89.43-121.97 |
| Propazine | 6.74-277.40 | 1.53-5.70 | ND | ND |
| Simazine | 5.00 | 4.55-37.79 | 1.61-12.5 | ND |
| Simetryn | ND | ND | ND | 77.83-102.77 |
| Terbuthylazine | ND | 2.15-35.54 | 8.8-10.3 | ND |
| Terbutryn | 5.00-182.87 | 0.82-73.46 | ND | ND |
| Prometryn | ND | ND | ND | 27.32-30.76 |

a-(Campo et al., 2013); b-(Köck-Schulmeyer et al., 2013); ND-not detected.
